# Fetal gestational age estimation using artificial intelligence on non-targeted ultrasound images and video

**DOI:** 10.1038/s41746-025-02024-z

**Published:** 2025-11-20

**Authors:** Martin Benson, Sacha Walton, Tom Hartley, Simon Meagher, Suresh Seshadri, Nicholas Sleep, Aris T. Papageorghiou

**Affiliations:** 1 GE HealthCare, Brunel House, Cardiff, CF24 0EB UK; 2 Monash Ultrasound for Women, Melbourne, VIC, Australia; 3 Mediscan Systems, Chennai, India; 4 Nuffield Department of Women’s and Reproductive Health and Oxford Maternal and Perinatal Health Institute, University of Oxford, Oxford, UK

**Keywords:** Computational biology and bioinformatics, Reproductive biology, Medical imaging, Biomarkers

## Abstract

We developed a deep learning model trained on over two million ultrasound images from 78,531 pregnancies from Australia, India, and the UK to estimate gestational age (GA) directly from any fetal ultrasound image, regardless of orientation. The model outputs both a GA estimate and an uncertainty value based on image quality. Independent validation on 36,762 ultrasound images from 742 fetuses showed a mean absolute error (MAE) of 1.7 days at 14–18 weeks and 2.8 days at 18–24 weeks, significantly outperforming traditional biometry (p < 0.001). In video analysis, the model achieved a median prediction time of 24 s and an MAE below 3 days across all trimesters. Performance was consistent across maternal body mass index (BMI) categories and geographic settings. This AI-based GA estimation method matches or exceeds gold-standard fetal biometry, reduces reliance on highly skilled sonologists, and offers the potential to improve access to prenatal care in resource-limited and underserved settings globally.

## Introduction

Accurate gestational age (GA) estimation is fundamental to prenatal care, guiding critical decisions that impact both maternal and fetal health. Knowledge of GA is essential as it influences the interpretation of fetal growth and well-being, the timing of medical interventions, and the planning and timing of birth.^1–3^.

Clinically, GA has been estimated using the last menstrual period (LMP) as a proxy for the time since conception. However, this method is associated with large errors due to assumptions about menstrual regularity and ovulation timing, coupled with the potential for inaccurate recall^1,4–7^. These limitations have driven the adoption of GA assessment using ultrasound-based fetal measurement. Ultrasound is a safe and widely used low-cost diagnostic technique and is a foundational aspect of prenatal care globally. Fetal ultrasound measurement is now considered the gold standard for GA assessment, particularly when performed early in the first trimester of gestation^8^. During this period, crown–rump length (CRL) measurements predict GA with a precision of 3–7 days^1–3,9–12^. Due to fetal curling with advancing pregnancy, the fetal CRL cannot be effectively measured after 14 weeks, and in the second and third trimester, a combination of other fetal measurements are used to determine estimated gestational age. The accuracy of GA determination by fetal biometry reduces with advancing gestation—it is ±7–10 days until 24 weeks and decreases to ±10 to 14 days between 24 and 28 weeks^1,3,13,14^. In the third trimester (greater than 28 weeks of gestation), ultrasound estimation is even less accurate, with previous studies reporting accuracy of ±21 to 30 days^1,3,13,14^.

These large errors, combined with the need for significant expertise on how to do ultrasound, has led to research into alternative methods. However, no approach has yet matched ultrasound for GA estimation across the full spectrum of pregnancy. Biomarkers, including human chorionic gonadotropin (hCG) and various metabolomic profiles, have shown some promise in early gestation but are hindered by inconsistencies, wide reference ranges, and limited windows of accuracy^15^. In low-resource settings, where late presentation to antenatal care is common, these limitations are particularly problematic.

Consequently, ultrasound remains the preferred modality for GA determination, and the World Health Organization (WHO) recommends that all pregnant women receive at least one ultrasound scan before 24 weeks of gestation^16^. However, this recommendation is aspirational in many low- and middle-income countries (LMICs): here the challenges center around the fact that accurate GA estimation through ultrasound requires the availability of the technology, but also expertise to obtain and interpret the necessary measurements. Obtaining precise biometric measurements demands considerable operator skill, time, and fetal cooperation, making it a burdensome task even in well-equipped settings^17^. This is compounded by the fact that in many LMICs, the first antenatal visit occurs late in pregnancy, which diminishes the accuracy of GA estimation. It is therefore desirable to develop methods for estimating GA which are easier, quicker, and require less operator skill to perform, and which can be applied at all gestations.

To address these challenges, we introduce a novel approach that trains a deep learning model on a very large and diverse dataset of ultrasound images. The data, stored during routine obstetric examinations along with corresponding gestational age data, are much larger and more diverse than those used previously: it contains data spanning three continents and is more than an order of magnitude larger than any used to develop similar models. Importantly, the model does not require images from biometry planes as used in most approaches defined by imaging protocols^2,18,19^ and can provide accurate GA estimates with minimal operator input. In other words, we sought to develop a model designed to work with images obtained without the need for specialized sonographic techniques, making it accessible to users with low levels of training. The model also includes an estimate of uncertainty, allowing for greater confidence in the results, particularly when images are suboptimal. This property of the model enables its potential use on ultrasound data obtained by a wider population, who have not been specially trained in sonography.

Our approach has the potential to democratize access to accurate GA estimation, particularly in settings where skilled sonographers are scarce. By enabling novice users to obtain reliable GA assessments, this technology could significantly enhance prenatal care in underserved regions, aligning with global health goals to improve maternal and child outcomes. In this paper, we present the development and validation of this AI-based model, demonstrating its superior performance compared to traditional biometry-based methods across a wide range of gestational ages and maternal characteristics. We also explore its potential for broader application, including its use in low-cost, portable ultrasound devices that could bring this critical diagnostic capability to even the most remote and resource-limited settings.

## Results

**Fig. 1:**
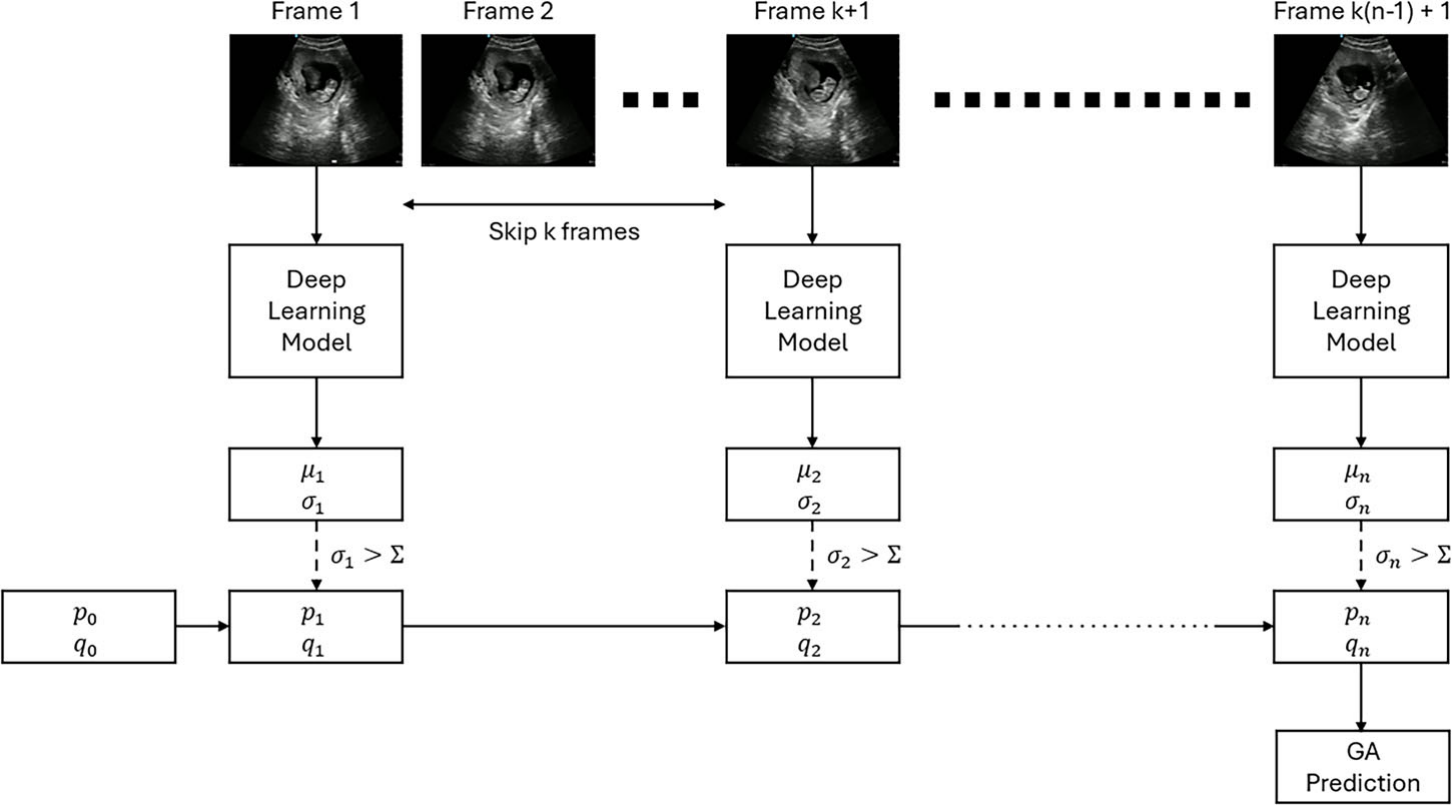
Kalman filtering process for generating predictions on video. In this figure, μ_1_, … ,μ_n_ and σ_1_, … ,σ_n_ denote sequences of mean and variance predictions from the model. p_1_, … ,p_n_ and q_1_, … ,q_n_ respectively denote sequences of state variables for the Kalman filter process. Σ is used to denote a threshold value on the variance predictions, below which no update is made.

As outlined above, the model was applied to test datasets containing two types of data: static images obtained from routine ultrasound examinations, and videos that simulate undirected ultrasound scanning (Fig. 1).

### Performance analysis on static images

The MAE of the model and biometry-based estimates (“Biometry measured”, as described in Comparison to biometric estimates) are summarized in Table 1. The AI model-based estimates were consistently more accurate than those obtained from biometry over the whole range of GAs from 10 to 36 weeks (Table 1). The superiority of GA estimation compared to that by biometry was strongly statistically significant from 14 to 24 weeks, and moderately significant over 24 weeks. The MAE expected for biometry-based estimates reported in the literature (“Biometry literature benchmark”) for that band are also provided in Table 1.

**Table 1:** Comparison of mean absolute errors by gestational age band

GA band (weeks)	Number of scans	Biometry literature benchmark MAE (days)	Biometry measured MAE (days)	IU ScanNav FetalCheck MAE (days)	MAE superiority to literature benchmark^*^-ve = improvement to benchmark	MAE superiority to measured −ve = improvement to benchmark	Superiority to measured *p* value^**^
10–14	190	2.8	-^***^	1.3	−1.5 (−54%)	-	-
14–18	100	3.0	3.4	1.7	−1.3 (−43%)	−1.7 (−50%)	<0.001
18–24	278	3.9	3.6	2.8	−1.1 (−28%)	−0.8 (−28%)	0.001
24–30	92	5.0	6.1	5.0	0.0 (0%)	−1.1 (−18%)	0.06
30–36	82	6.8	5.7	4.7	−2.1 (−31%)	−1.0 (−18%)	0.08

*GA* gestational age, *MAE* mean absolute error.

*Absolute difference is reported, followed by relative difference in brackets.

^*^^*^Calculated via the Wilcoxon signed-rank test.

***Since CRL-based GA is by definition correct in this band, the measured value is not reported.

Subanalysis of MAE of the AI model and biometry-based estimates by country demonstrates that model estimates were at least as accurate as those obtained from biometry in all scanning countries (Table 2), with results significantly more accurate for the UK and Australia. The lack of significance among scans in the India subset appears to be driven by a lower-than-expected error of the biometry-based estimates obtained during weeks 18+, which are around 1 day lower than the levels that the literature implies they should be. The cause of this is unknown, but could, for example, be caused by operators having sight of the gold standard GA during scanning and expected value bias—a behavior commonly observed^20^. Analysis of MAE by maternal body mass index (BMI) categories suggested stable performance of the AI model (Table 3).

**Table 2:** Comparison of mean absolute error by scanning country

Scanning country	Number of scans	Biometry measured MAE (days)	IU ScanNav FetalCheck MAE (days)	MAE superiority to measured	Superiority to measured *p* value*
UK	98	2.4	1.5	−0.9 (−38%)	<0.001
India	344	5.4	3.5	−1.9 (−35%)	0.14
Australia	300	3.7	2.2	−1.5 (−41%)	<0.001

*MAE* mean absolute error.

^*^Calculated via the Wilcoxon signed-rank test.

Subanalysis of MAE by maternal BMI shows that model estimates are at least as accurate as those obtained from biometry for all bands of maternal BMI (Table 3).

**Table 3:** Comparison of mean absolute error by maternal body mass index

Maternal BMI	Number of scans	Biometry measured MAE (days)	IU ScanNav FetalCheck MAE (days)	MAE superiority to measured	Superiority to measured *p* value
<25	107	5.9	3.5	−2.4 (−40%)	0.51
25 to 30	129	6.4	3.8	−2.6 (−40%)	0.12
>30	108	3.8	3.2	−0.6 (−15%)	0.22

*MAE* mean absolute error, *BMI* body mass index.

**Fig. 2:**
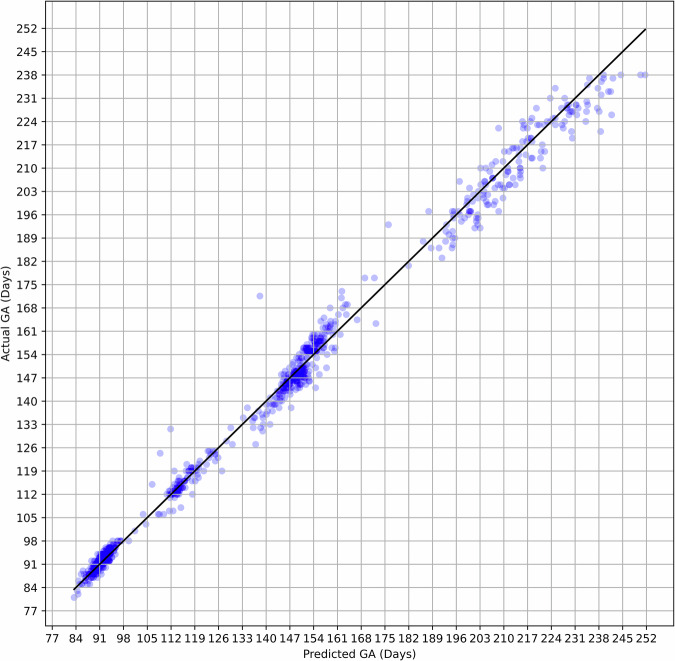
Predicted versus actual gestational age. Scatter plot comparing the gestational age (GA) predicted by the AI model with the actual GA based on gold standard crown–rump length dating (plus time elapsed). Each point represents a prediction for a single case. The data points cluster closely and uniformly around the diagonal line, demonstrating high concordance between predicted and actual values.

**Fig. 3:**
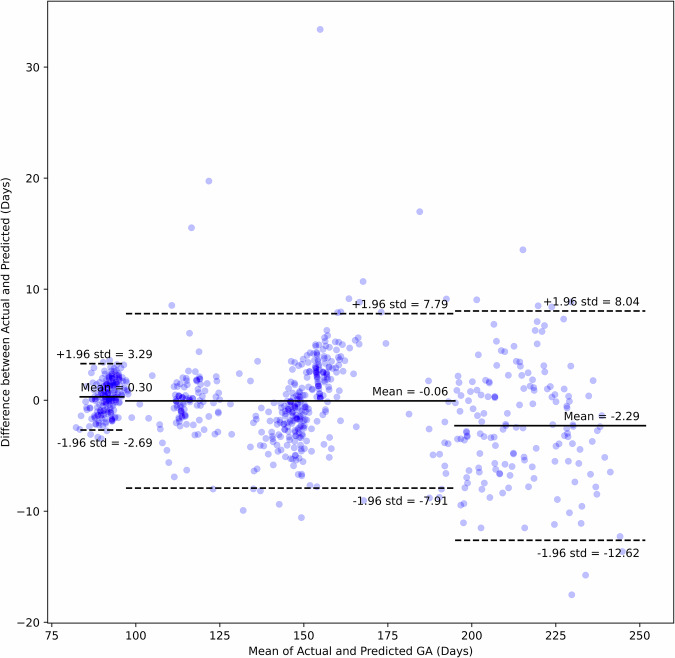
Bland–Altman plot of predicted versus actual gestational age. This plot illustrates the agreement between the AI-predicted gestational age and the actual (gold standard) gestational age.

The relationship between the predicted gestational age generated by the model and the corresponding ground-truth GA values are shown in Fig. 2. Visual assessment of the accuracy, precision, and potential biases of the model’s predictions demonstrates that data points are clustered around the diagonal line representing perfect prediction. The Bland–Altman plot represented in Fig. 3 offers a complementary perspective on the relationship between predicted gestational age and corresponding actual GA values. It confirms a high degree of correspondence between the predicted and actual values, and also shows, in common with other approaches to estimating GA, that error magnitudes increase with GA.

### Performance analysis on videos

The MAE of the model estimates on the video test dataset have been calculated and summarized within bands of (actual) GA, and are reported in Table 4. They show that the model estimates are more accurate than would be expected to be obtained from biometric measurement in all trimesters.

**Table 4:** Mean absolute error of the estimates obtained from the video

GA band	Number of scans	Expected biometry MAE (days)	IU ScanNav FetalCheck MAE (days)	MAE superiority to expected
10–14 Weeks	36	2.8	2.5	−0.3 (−11%)
14–27 Weeks	58	3.9	3.7	−0.2 (−5%)
27–34 Weeks	5	5.4	2.6	−2.8 (−52%)

*GA* gestational age, *MAE* mean absolute error.

A scatter plot and Bland–Altman plot of the relationship between predicted and actual GA values can be found in the Supplementary Appendix.

**Fig. 4:**
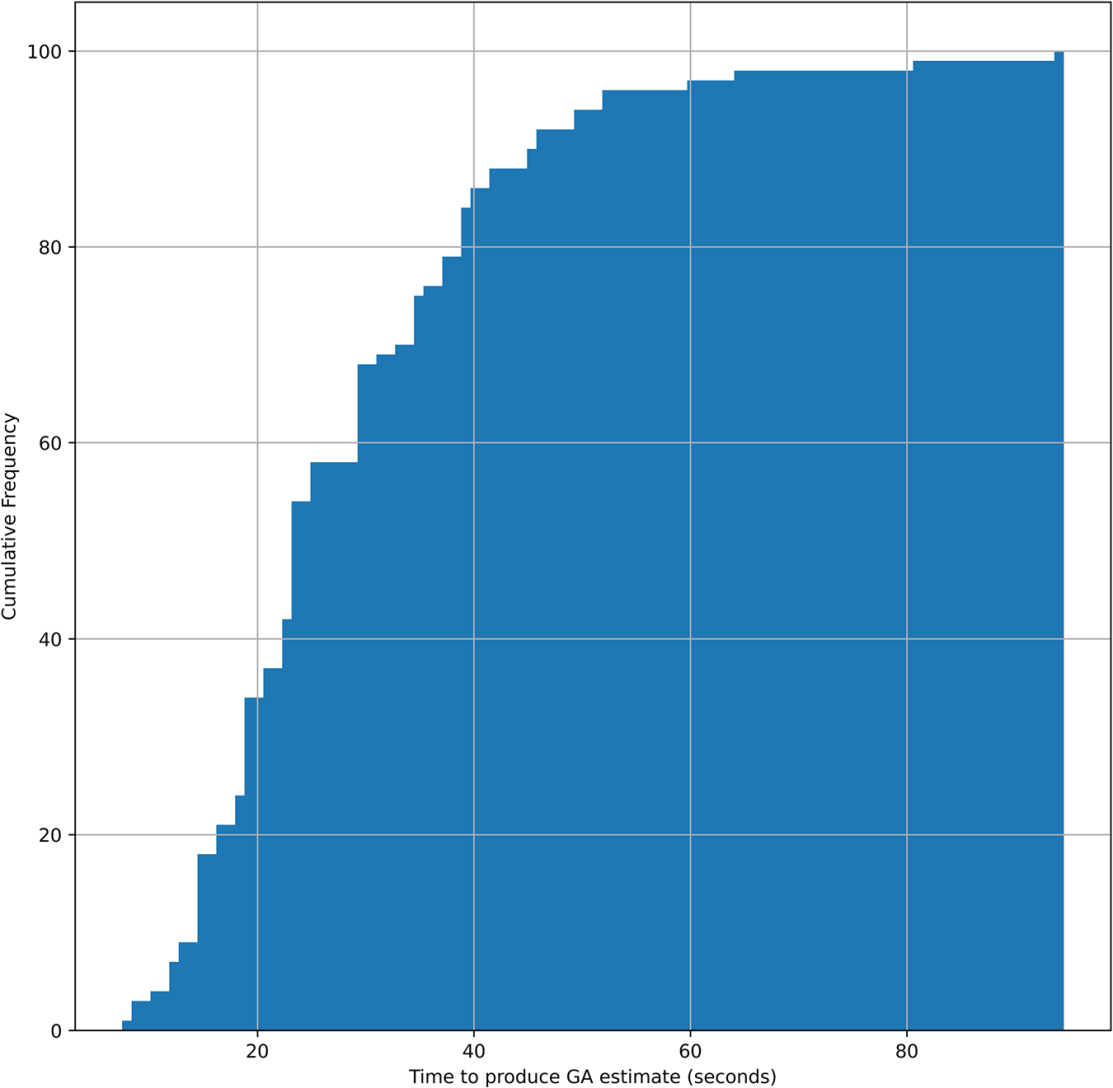
Cumulative distribution of the time taken to generate gestational age prediction. The cumulative distribution of time (in seconds) required by the AI model to produce a sufficiently confident gestational age (GA) estimate from video data. The x-axis represents elapsed time since the start of the video, and the y-axis indicates the proportion of cases for which the model had already generated a prediction. The model typically produces estimates rapidly, with a median prediction time of 24 s, and over 95% of predictions are completed within 60 s. This highlights the efficiency of the model in real-time scanning contexts.

The amount of time needed to generate a sufficiently confident GA estimate varies based on the level of useful information encountered within the frames of each video. Figure 4 shows the cumulative distribution of the time needed to produce predictions on the test set, demonstrating that in 95% of cases less than a minute of video is needed. The median time to produce an estimate is 24 s. Example videos across gestational age, including a failure case, are given in the Supplementary Videos 1–4.

To assess the impact that re-shuffling the 5-s video snippets within the videos has on model predictions, a GA estimate was produced for shuffled and re-shuffled versions of the same video. This resulted in re-shuffled videos presenting frames to the model in a different order, but also, as noted above, some frames that were different from the original shuffled video. The difference in the estimates obtained from each pair of videos was less than 3 days in around 90% of cases. This is a relatively small movement in relation to the average magnitude of error in the estimates, suggesting that the accuracy of model estimates was not strongly dependent on the order in which fetal anatomy is scanned.

## Discussion

The main contributions of this paper are to: (i) Introduce a method of training a Deep Learning model from very large retrospective ultrasound image datasets capable of outputting both a GA estimate and a well-calibrated estimate of the level of uncertainty inherent in that estimate, (ii) Introduce a method for applying such a model to a succession of ultrasound images obtained from a video sequence or collection of static images, by applying a static 1D Kalman filter to its outputs and (iii) Train such a model on a large, very diverse dataset of ultrasound images, containing data on over 75k fetuses, and sourced from scanning centers spanning three continents.

Our results show that the accuracy of the outputs of this model is superior to estimates obtained via current standard clinical practice (biometric measurement), both at an overall level and in all key subset analyses that were performed.

While direct comparisons to other results presented in the literature are made difficult by differences in data, scanning approach, and methods of analysis, we consider that the results that we have presented are competitive with those of ref. ^21^, who developed a deep learning neural network model trained on a dataset of 1968 subjects to estimate GA from short ultrasound videos, some of which were acquired via a blind sweep scanning protocol, and some of which were “fly-to” videos targeting biometry planes. Their model achieved MAE values of approximately 1.92 days (14–19 weeks), 2.78 days (20–25 weeks), 2.97 days (26–31 weeks), and 3.07 days (32–37 weeks) when measured on videos obtained from trained sonographers following a blind sweep protocol. They further demonstrated that accuracy was non-inferior to biometry-based estimates when applied to videos obtained from novices following the blind sweep protocol (though lower accuracy than that performed by a trained sonographer). The methods we describe in this paper have the benefit of being readily applicable to large retrospective image archives, rather than requiring video data for model training.

Maraci et al. ^22^ describe a method for estimating GA by applying deep learning to images of the trans-cerebellar view of the fetal head to produce an automated TCD measurement that can then be a GA estimate via standard reference charts. Their model was trained on a dataset of 500 ultrasound images of fetuses for between 16 and 26 weeks GA and yielded MAE values of ~5.4 days, though with respect to a GA estimate obtained through manual TCD measurement, which is not the gold-standard approach. Finally, ref. ^23^ describes a slightly more complex approach which uses deep learning to analyse images from multiple biometric planes (TV, AC, and FL) to estimate GA directly. They utilized a dataset of 3809 subjects and reported MAE values of 2.6 days during 14–19 weeks of pregnancy, 3.1 days (20–25 weeks), 3.3 days (26–31 weeks), and 4.6 days (32–37 weeks). A drawback of both of the latter two approaches is that it requires the operator to acquire images from particular planes, meaning that they are not applicable to scanning by novices.

A limitation of the analysis presented is that it does not quantify how accurate the model is when applied directly to videos of scans obtained by novice users. A natural next step in enhancing our understanding of the performance of our method in a real-world setting will be to perform prospective study in which novice users obtain GA estimates using it and then to analyse the results of that.

We consider that the methods presented represent progress towards democratizing the use of ultrasound in clinical decision making, through developing automated systems that are able to interpret ultrasound data that is acquired without requiring sonographic training. Such systems could be operated by a wider set of medical practitioners, including, for example, General Practitioners and midwives. This could drive improvements in clinical care in LMICs, especially, but may also enhance efficiency and patient outcomes in developed countries too.

## Methods

We trained a deep learning model that estimates fetal GA directly from ultrasound images, without requiring measurement information or the acquisition of specific views of the fetus. The model outputs not just an estimate of the GA, but also the level of uncertainty that is inherent in the estimate. The level of uncertainty that it reports varies from image to image and depends upon the following factors:

i. The amount of useful information within the image (some images, for example, when the probe is not pointing at the fetus, are entirely unsuitable for GA estimation).
ii. The extent to which the image is of a type that is well represented in the training data. The further out-of-distribution an image is, the greater the uncertainty in the estimate will be.

Model training was undertaken using fetal ultrasound image data, which was collected from multiple centers in Australia, India, and the UK, creating a diverse dataset. A stratified sampling strategy was applied to the training data based on GA bands and ultrasound probe types, to ensure robust performance across various clinical scenarios.

Model validation was conducted on several independent retrospective datasets, assessing accuracy of the approach in comparison to GA estimates derived from clinical biometric measurements like biparietal diameter and head circumference, to evaluate the model’s efficacy throughout pregnancy and across different maternal BMI categories.

Both training and validation datasets were annotated with gold standard GA estimates, taken during routine clinical practice. The analysis was done entirely offline, on retrospective datasets.

### Gold standard for GA

Throughout the development and validation analysis, the “ground-truth” GA value that the model output was compared against was computed from a previous CRL measurement taken between 9 + 0 and 13 + 6 weeks. More specifically:

- At all centers, CRL measurements were taken by expert and quality-controlled sonologists.
- That measurement was converted to a GA estimate as per^9^. This GA estimate is related to the day on which the CRL measurement was taken.
- The GA estimate was then adjusted forward to the date of the ultrasound examination (when the relevant image was captured, in many cases in a subsequent examination to the one in which CRL measurement was performed), by adding the number of days elapsed since the CRL measurement was taken.

### Datasets

For model development, we collated a large, multicenter anonymised dataset of ultrasound imaging data from six centers in Australia, India, and the UK. These datasets (Table 5) contain imaging records obtained during routine clinical scanning, together with associated information on gestational age and a limited amount of maternal demographic information.

**Table 5:** Summary of retrospective ultrasound image data obtained during routine clinical scanning, collated from multiple centers in India, Australia, and the UK

Data Source	Images (*n*)	Dataset description	Route	
Australia	9,104,090	Imaging centers, Melbourne, Australia.	TA and TV	GE Voluson E6, E8 and E10.
India	1,028,534	Hospital-based, Chennai, India.	TA and TV	GE Voluson E6, E7, E10, P8, S6 and Mindray Resona 7.
UK	639,618	Imaging Center, London, UK.	TA	GE Voluson E10 and Expert 22.

*TA* transabdominal, *TV* transvaginal.

To ensure robust model performance (including mitigating the possible impact of confounding effects), and in support of an efficient training process, a stratified sample of the available data was used to develop the model. The strata used when sampling were:

- Data source
- GA (in 4-week bands)
- Ultrasound probe type (TA/TV)

The data over those strata were balanced by randomly sampling varying numbers of images per subject. No exclusions were made from the development data. It therefore contains (in approximately their natural frequencies) singleton and multiple pregnancies, as well as congenital abnormalities. This a priori decision was considered to be useful from the perspective of external validity and model performance under a wide range of circumstances.

The sampled data were split, using computerized randomization, between training and validation sets in a 90:10 proportion in order to yield a robust validation volume while retaining as much data for model training as possible. The unit of randomization was the individual woman, so that all images pertaining to any given subject were assigned to either the training set or the validation set. The validation set was used during the model development process to optimize hyperparameters and to confirm the model’s ability to generalize.

Following sampling and assignment, the data volumes used to develop the model were as detailed in Table 6. Note that the total number of images (but not the number of data subjects) used is lower than the total available due to the sampling process described above, which sampled only a random sample of the images for each subject.

**Table 6:** The data volumes used for developing the model

Data source	Training set		Validation Set	
	Number of images	Number of subjects	Number of images	Number of subjects
India	610,208	29,094	67,386	3215
Australia	1,149,244	41,391	127,627	4607
UK	240,877	8046	25,258	896
Total	2,000,329	78,531	220,271	8718

A subsample of the retrospective image data were also set aside as a test set, as described in the performance analysis section. The sampling was performed at a subject level so that no fetus that was present in the development data was present in the test dataset, and vice versa.

A further test dataset was also sourced from a different UK imaging center to the one which provided the retrospective image data, comprising a sample of videos covering entire routine ultrasound scans Table 7. The dataset contains around 100 scans and covers all trimesters. More details on the video test set may be found in the performance analysis section.

**Table 7:** Summary of retrospective image data for model validation

GA Band	Number of data subjects
(10,14]	193
(14,18]	98
(18,22]	184
(22,26]	100
(26–30]	84
(30–34]	83
Total	742

*GA* gestational age.

As we used real-world data, image datasets contained images of varying resolutions, formats, and colors. Therefore, all were resized to 384 × 576 pixel greyscale PNG images in a pre-processing step. This resolution was chosen to deliver the desired runtime performance characteristics for the target model architecture.

### Deep learning model

The model training and prediction pipelines were implemented in Python using Pytorch v1.9.0. Model training was performed on a GPU cluster containing 16 Nvidia RTX A4000 GPUs.

A deep learning^24^ model was used to produce GA estimates from ultrasound images trained via supervised learning—a process by which the model is optimized to generate estimates that are as close as possible to those provided in the training dataset. Deep Learning has demonstrated state of the art performance in computer vision tasks since 2012. ^25^ and now dominates the field, making it a natural approach for this task.

One challenge in producing GA estimates based on ultrasound images taken by novices is that images may be unsuitable for this purpose (as an extreme example, images may not show a fetus at all). To overcome this problem, we designed the model to report the level of uncertainty in GA prediction. To do this, we follow the approach described by ref. ^26^ to construct a neural network comprising the following elements:

- A trunk network ${f}_{\mathrm{trunk}}:\left(X,\,{\Theta }_{z}\right)\to {{\rm{{\mathbb{R}}}}}^{d}$ which, given parameters ${\theta }_{z}\epsilon {\Theta }_{z}$ maps an image $x\epsilon X$ to a $d$ dimensional representation vector. We employed a ConvNeXt^27^ architecture (“small” variant) for ${f}_{\mathrm{trunk}}$ with $d=768$
- A mean prediction head $\mu :\left({{\mathbb{R}}}^{d},\,{\Theta }_{\mu }\right){\mathbb{\to }}{\mathbb{R}}$ which, given parameters ${\theta }_{\mu }\epsilon {\Theta }_{\mu }$ maps a representation vector $v\epsilon {{\mathbb{R}}}^{d}$ to an estimate of the mean of a normal distribution describing the target variable
- A variance prediction head $\sigma :\left({{\mathbb{R}}}^{d},\,{\Theta }_{\sigma }\right)\to {{\mathbb{R}}}^{+}$ which, given parameters ${\theta }_{\sigma }\epsilon {\Theta }_{\sigma }$ maps a representation vector $v\epsilon {{\mathbb{R}}}^{d}$ to an estimate of the sigma parameter of a normal distribution describing the target variable

Since they are non-negative, we modeled the GA values in log space by taking the natural logarithm of the labels.

The parameter optimization process sought to minimise

1 $${\mathcal{L}}:=\mathop{\sum }\limits_{(x,y)\epsilon {\mathcal{D}}}\frac{{|y-\mu ({f}_{trunk}(x))|}_{2}^{2}}{2}-{\text{ln}}\,{\mathcal{N}}(y|\lfloor \mu ({f}_{trunk}(x))\rfloor ,\sigma (\lfloor \,{f}_{trunk}(x)\rfloor ))$$

Where

- ${\mathcal{D}}$ denotes the dataset of image $x$ and label $y$ pairs
- ${\left|\cdot \right|}_{2}$ denotes the ${l}^{2}$-norm
- ${\mathcal{N}}$ denotes the PDF of a standard normal distribution
- $\lfloor \cdot \rfloor$ denotes a stop-gradient operation

Despite the stop-gradient operations applied in this approach, we found that phased model training delivers superior results:

- Phase 1 – freeze the variance prediction head, optimizing only parameters in the trunk and mean prediction head.
- Phase 2 – Finetune from the Phase 1 model, with all parameters unfrozen.

Each phase was trained for 100 epochs, using the Adam optimizer^28^ with $\varepsilon ={10}^{-8}$, ${\beta }_{1}=0.9$ and ${\beta }_{2}=0.999$. Learning rate was set on a two-phase schedule: a linear warm-up phase increasing it from 0 to a maximum value over the first 10 epochs and then decreasing it again to 0 over a cosine annealing schedule over the following 90 epochs. The maximum learning rate that was applied was derived via the process described in ref. ^29^.

Training data were augmented during mini-batch preparation to enhance diversity and encourage better model generalization. A variant of the RandAugment^30^ approach to augmentation was used. The main variations made were to select a set of transformations better suited to ultrasound images, by which a random set of transformations was selected per image. The list of available transformations was tailored towards being appropriate for ultrasound images, and is as follows: rotation, re-scaling, horizontal flip, blur, brightness & contrast jitter, pixel-wise multiplicative noise, and grid distortion. We randomized the *N* parameter to a value in a chosen range, rather than selecting a specific value.

### Applying to video data

Typically, several images of the fetus are obtained during an ultrasound examination, and in real-world imaging, this is in the form of a real-time video. To enable applicability to video clips, our method was designed to generate more precise estimates when multiple images of a given fetus are available, by applying a static 1D Kalman filter to the estimates (and corresponding uncertainties) that are obtained by applying the deep learning model to the individual images. Given a sequence of input images ${x}_{1},\cdots ,{x}_{n}$ we apply to model to gain sequences of mean and variance predictions, ${\mu }_{1},\cdots ,{\mu }_{n}$ and ${\sigma }_{1},\cdots ,{\sigma }_{n}$ respectively. The Kalman filtering process sequentially updates state variables representing the mean and standard deviation of the process, which we denote ${p}_{1},\cdots ,{p}_{n}$ and ${q}_{1},\cdots ,{q}_{n}$, via the following Eqs. (2) to (4) ($1\le i\le n$):

2 $${K}_{i}=\frac{{p}_{i-1}^{2}}{{p}_{i-1}^{2}+{\sigma }_{i}^{2}}$$

3 $${p}_{i}={p}_{i-1}+{K}_{i}\left({\mu }_{i}-{p}_{i-1}\right)$$

4 $${q}_{i}=\sqrt{\left(1-{K}_{i}\right){q}_{i-1}^{2}+\varepsilon }$$

Where $\varepsilon$ is process noise (we set $\varepsilon =0.001$). We initialize the state variables with ${p}_{0}=4.94$ (i.e., corresponding to a GA of 140 days) and ${q}_{0}=0.35$ (i.e., corresponding to a 95% prediction interval of (70 days, 278 days)).

Underpinning the Kalman filtering algorithm is an assumption that noise is uncorrelated over observations, but this is unlikely to be true where the observations arise from successive frames of a video. Subsampling the frames helps to reduce this correlation, and in our implementation, we sample 1 frame per second.

Videos of real-world scanning often include periods in which the ultrasound image does not actually show the fetus (for example, before the probe is placed on the maternal abdomen), and we find that performance is improved by screening out such frames from the Kalman filtering process. We find that applying a threshold to the ${\sigma }_{i}$ values works well as a heuristic for this (we exclude frames where ${\sigma }_{i} > \Sigma =0.1$).

This process is shown in Fig. 1. 

### Performance analysis

Analysis of model performance was undertaken by analysis of retrospective data that were wholly independent of the data used in model development. GA estimates calculated by the AI model were compared to GA estimates according to fetal biometric measurement at the same ultrasound examination, which represents clinical best practice. This comparison was made using mean absolute error (MAE).

Two test datasets were used in the analysis:

1. Retrospective image data: Comprising a large, diverse set of static image sets sampled from the retrospective image archives that were used for model training, but containing an entirely independent set of subjects.
2. Retrospective video data: Comprising a smaller sample of ultrasound videos, that were created by splicing together small random subsegments of full-length fetal scans in a random order to approximate scans conducted in an undirected manner. The ultrasound scans were obtained from the UK imaging center (different from the one used to source the development data)

### Assessment of performance on static images

This component of the analysis compared the accuracy of the model estimates to those obtained from biometric measurement versus the gold standard. It was conducted on a set of images, 36,762 images from 742 fetuses that were stored in patient records during routine ultrasound examinations. Stratification and sample size estimation was conducted to ensure that a robust assessment of model accuracy could be made over a number of key dimensions, while working within constraints imposed by data availability. The dimensions considered were:

- Stage of pregnancy
- Maternal BMI
- Country in which the scan is conducted

The structure of the static image test set is summarized in Table 1. Sample sizes were estimated to enable detection of a 1-day difference in MAE at 90% confidence level for all subsets of interest.

The subjects sampled into the test dataset were entirely independent of those used for model development—no data from any of subject included in the test dataset was used at all during the development process.

### Assessment of performance on videos

This component of the analysis aimed to confirm that the model estimates are accurate when calculated from videos of scans that were obtained in an undirected fashion. It further aimed to establish that the results obtained from the model do not depend heavily on the particular order in which fetal anatomy happens to be scanned. A test dataset was created that consists of videos of ultrasound scanning, designed to simulate undirected acquisition (for example, scanning without sight of the images). To achieve this, 99 full-length videos of a routine ultrasound examinations and where actual GA was known (based on previous CRL, the gold standard) were used. From these 99 videos, we created 99 3-min videos via the following process:

- For each scan, randomly select 36 non-overlapping 5-s subsegments of the video
- For each scan, randomly shuffle the order of the subsegments
- Concatenate together the shuffled subsegments into a 3-min clip

For each of these videos, we also repeated steps 2 and 3 to produce another video having the same content but with the frames in a different order.

During the process of video analysis, the model assesses each frame it sees to determine whether it contains a frames containing useful information and the model only utilizes frames that contain such information (The full-length video data that we use are real-world data and contain segments of 3D/4D scanning and pulse-wave Doppler scanning, for example, which are not useful to the model and these were not used in training). Because the model outputs estimates once it generates a sufficiently confident result, then ignoring the rest of the video, this means that the re-shuffling process may result in different parts of fetal anatomy being presented to the model during the period that it analyses.

We have also analysed the time taken by the algorithm to produce an estimate on each of these videos, and report the cumulative distribution.

### Comparison to biometric estimates

In current clinical practice, GA estimation is undertaken using ultrasound-measured fetal biometry. GA estimates by biometry were calculated from measurements of the fetal biparietal diameter (BPD), head circumference (HC), abdominal circumference (AC), and femur length (FL) associated with the scans and based on the following formula (5)^14^:

5 $$G{A}_{{d}{a}{y}{s}}=7\times \left(10.85+\left(0.0006\times HC\times FL\right)+\left(0.067\times BPD\right)+\left(0.0168\times AC\right)\right)$$

For scans that were conducted prior to week 14, the clinical gold-standard approach to estimating GA is via CRL measurement, as described above. This means that the biometric estimates are, by definition, correct and comparison of model estimates are therefore not feasible. Instead, we compare to an accuracy benchmark^12^, which quantifies the error in CRL-based estimates relative to GAs calculated from conception dates that are known with certainty.

## Supplementary information

Supplementary Appendix R3

Supplementary movie 1

Supplementary movie 2

Supplementary movie 3

Supplementary movie 4

## Data Availability

The data used for this work is subject to data-sharing restrictions preventing broad availability. Data access for non-commercial purposes can be requested via email to the first author (Martin.Benson@gehealthcare.com) with a detailed analysis plan. Access may be granted on a controlled basis following consent from data owners.

## Code availability

The code supporting this work is proprietary to GE HealthCare. A minimal version of this code, excluding proprietary components, can be made available upon a licensing agreement. For inquiries, please contact the corresponding author.
