# Supplementary material for: Fetal gestational age estimation using artificial intelligence on non-targeted ultrasound images and video
Source: NPJ Digit Med. 2025 Nov 20;8:700. doi: 10.1038/s41746-025-02024-z (PMC12635104; doi:10.1038/s41746-025-02024-z)
Supplement: Supplementary file 1 — Supplementary Appendix R3 [file 41746_2025_2024_MOESM1_ESM.pdf]

Supplementary Appendix

Supplementary Figure 1. Predicted vs Actual GA Scatter Plot *based on* video data

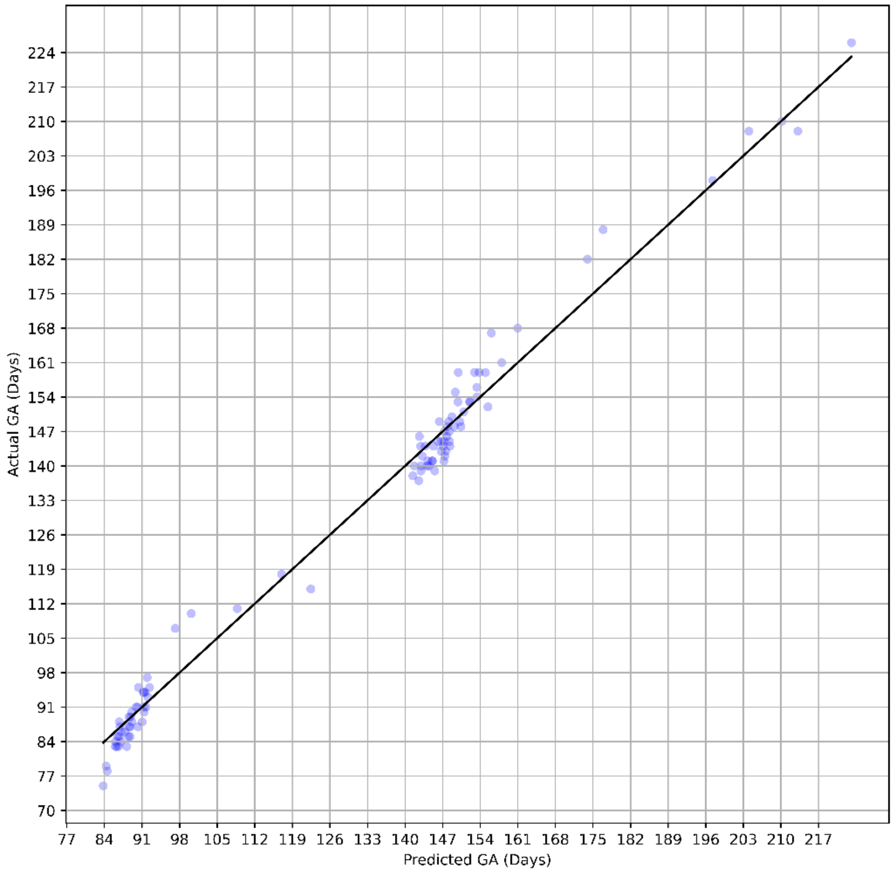

Supplementary Figure 2. Predicted vs Actual GA Bland-Altman Plot based on video data

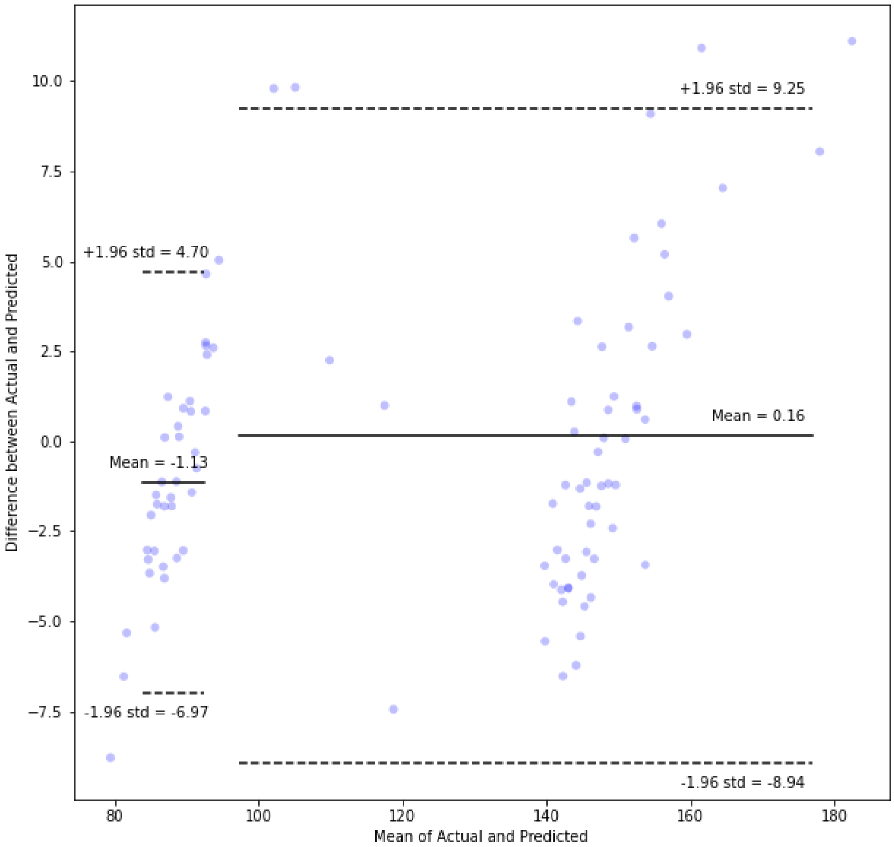

Supplementary Movie 1: Successful automated gestational age estimation at 12_{+0} weeks of gestation
Supplementary Movie 2: Successful automated gestational age estimation at 16_{+3} weeks of gestation
Supplementary Movie 3: Successful automated gestational age estimation at 23_{+0} weeks of gestation
Supplementary Movie 4: Unsuccessful automated gestational age estimation
